# Comparative tumor promotion assessment of e‐cigarette and cigarettes using the in vitro Bhas 42 cell transformation assay

**DOI:** 10.1002/em.22091

**Published:** 2017-04-26

**Authors:** Damien Breheny, Oluwatobiloba Oke, Kamala Pant, Marianna Gaça, G. Umbezeiro

**Affiliations:** ^1^ British American Tobacco, Research and DevelopmentSouthamptonUK; ^2^ Department of Genetic ToxicologyBioreliance CorporationRockvilleMaryland

**Keywords:** e‐cigarettes, tobacco smoke, in vitro, carcinogenesis, risk assessment, nongenotoxic

## Abstract

In vitro cell transformation assays (CTA) are used to assess the carcinogenic potential of chemicals and complex mixtures and can detect nongenotoxic as well as genotoxic carcinogens. The Bhas 42 CTA has been developed with both initiation and promotion protocols to distinguish between these two carcinogen classes. Cigarette smoke is known to be carcinogenic and is positive in in vitro genotoxicity assays. Cigarette smoke also contains nongenotoxic carcinogens and is a tumour promoter and cocarcinogen in vivo. We have combined a suite of in vitro assays to compare the relative biological effects of new categories of tobacco and nicotine products with traditional cigarettes. The Bhas promotion assay has been included in this test battery to provide an in vitro surrogate for detecting tumor promoters. The activity of an electronic cigarette (e‐cigarette; Vype ePen) was compared to that of a reference cigarette (3R4F) in the promotion assay, using total particulate matter (TPM)/aerosol collected matter (ACM) and aqueous extracts (AqE) of product aerosol emissions. 3R4F TPM was positive in this assay at concentrations ≥6 µg/mL, while e‐cigarette ACM did not have any promoter activity. AqE was found to be a lesssuitable test matrix in this assay due to high cytotoxicity. This is the first study to use the Bhas assay to compare tobacco and nicotine products and demonstrates the potential for its future application as part of a product assessment framework. These data add to growing evidence suggesting that e‐cigarettes may provide a safer alternative to traditional cigarettes. Environ. Mol. Mutagen. 58:190–198, 2017. © 2017 The Authors. Environmental and Molecular Mutagenesis Published by Wiley Periodicals, Inc.

## INTRODUCTION

Cigarette smoke is a highly complex mixture of more than 6,000 chemicals [Rodgman and Perfetti, 2013]. Of these compounds, approximately 150 are known toxicants, including both genotoxic and nongenotoxic carcinogens. Traditionally, in vitro genotoxicity assays such as the *Salmonella typhimurium* mutation (Ames) and micronucleus assays have been used extensively to assess tobacco products [Aufderheide and Gressmann, 2008; DeMarini et al., 2008; Thorne et al., 2015]. However, by their very nature they only focus on initiating events, i.e., DNA damage. To form a comprehensive understanding of potential carcinogenic risk, there is a need to also investigate later cancer related‐endpoints, including tumor promotion by nongenotoxic carcinogens that act by various other means such as the disruption of cellular communication and signal transduction pathways.

In vitro cell transformation assays (CTA) enable the detection of both genotoxic and nongenotoxic compounds. The Bhas 42 assay is one such example, which has the additional capability of distinguishing between these two classes of carcinogenic compounds [Sasaki et al., 1990; Ohmori et al., 2004, 2005]. The potential initiator and/or promoter activity of test compounds can be detected via two separate protocols. In recent years, this assay has undergone a number of international validation studies and is now the subject of an OCED guidance document [Organisation for Economic and Cooperative Development, 2016]. Weisensee et al. [2013] and Han et al. [2016] have shown that Bhas 42 CTA could be useful in assessing the promoter activity of complex mixtures such as cigarette smoke. Their work showed that total particulate matter (TPM) from a reference cigarette induced cell transformation in a concentration‐dependent manner. These findings support long‐established knowledge of the tumor promoter activity of cigarette smoke “tar” in rodent skin painting studies [Roe et al., 1959] and demonstrate the suitability of this assay as an in vitro alternative for this application.

While conventional cigarettes generate complex aerosols that are known to contribute to diseases such as lung cancer in smokers, electronic cigarettes (e‐cigarettes) may provide a much safer alternative due to the less complex aerosol that they produce. The use of e‐cigarettes has increased significantly in recent years. Public Health England recently reported that e‐cigarettes were “95% less harmful to health than conventional cigarettes” and could be a means for smokers to quit or reduce their consumption of cigarettes [Public Health England, 2015]. The Royal College of Physicians have recently stated that “the hazard to health arising from long‐term vapour inhalation from the e‐cigarettes available today is unlikely to exceed 5% of the harm from smoking tobacco” [Royal College of Physicians, 2016].

We have recently reported that the levels of toxicant aerosol emissions from a commercially available e‐cigarette (Vype ePen) are 92–99% lower on a per‐puff basis than those from a reference cigarette (3R4F) [Margham et al., 2016]. While the greatly reduced chemical profile in e‐cigarette aerosols certainly suggests much less activity than conventional cigarettes in toxicological assays, direct evidence of this has been limited to date. We have shown in vitro, that ePen e‐cigarette aerosols are nonresponsive in mutation and DNA damage assays compared to 3R4F reference cigarette smoke which demonstrated strong mutagenic and DNA damage responses under the same experimental conditions [Thorne et al., 2016, 2017]. ePen e‐cigarette aerosol extracts also conferred much lower oxidative stress in vitro using human lung cells than those from a 3R4F reference cigarette [Taylor et al., 2016]. It must be noted that e‐cigarettes in general can vary greatly in their aerosol emission as a result of device characteristics and the formulation of the liquid, which may or may not contain nicotine [Farsalinos et al., 2016].

The purpose of this study was to compare the promoter activities of a traditional conventional cigarette versus a commercially available e‐cigarette as measured by the Bhas promotion CTA in both the particulate and vapour phases. TPM generated from 3R4F reference cigarettes and aerosol collected matter (ACM) from Vype ePen e‐cigarette enabled the assessment of the particulate fraction of the cigarette aerosol and the corresponding nongaseous phase of the e‐cigarette aerosol, respectively (e‐cigarette aersols do not contain particulates, but rather aerosolized propylene glycol and vegetable glycerine). Aerosol aqueous extracts (AqE) generated from both product types provided the assessment of the particulate and water‐soluble components of the vapor phase. Previous studies assessing cigarette smoke in the Bhas 42 assay have used TPM prepared using the ISO puffing regime standard 3308 [International Organization for Standardization, 2000]. For this study, a more intense smoking regime Health Canada Intense (HCI) was used in preparing the TPM, ACM, and AqE.

## MATERIALS AND METHODS

### Chemicals and Reagents

All chemicals and reagents used were obtained from Sigma‐Aldrich (Gillingham, UK) unless otherwise stated.

### Test Products

The 3R4F reference cigarette (University of Kentucky, Kentucky) was used in this study. This king‐size product contains US blended tobacco with a cellulose acetate filter having a International Organisation for Standardisation (ISO) tar yield of 9.4 mg. Prior to the production of TPM or AqE, cigarettes were conditioned for at least 48 hr at 22 ± 1°C and 60 ± 3% relative humidity in accordance with ISO 3402 [International Organization for Standardization, 1999].

**Figure 1:**
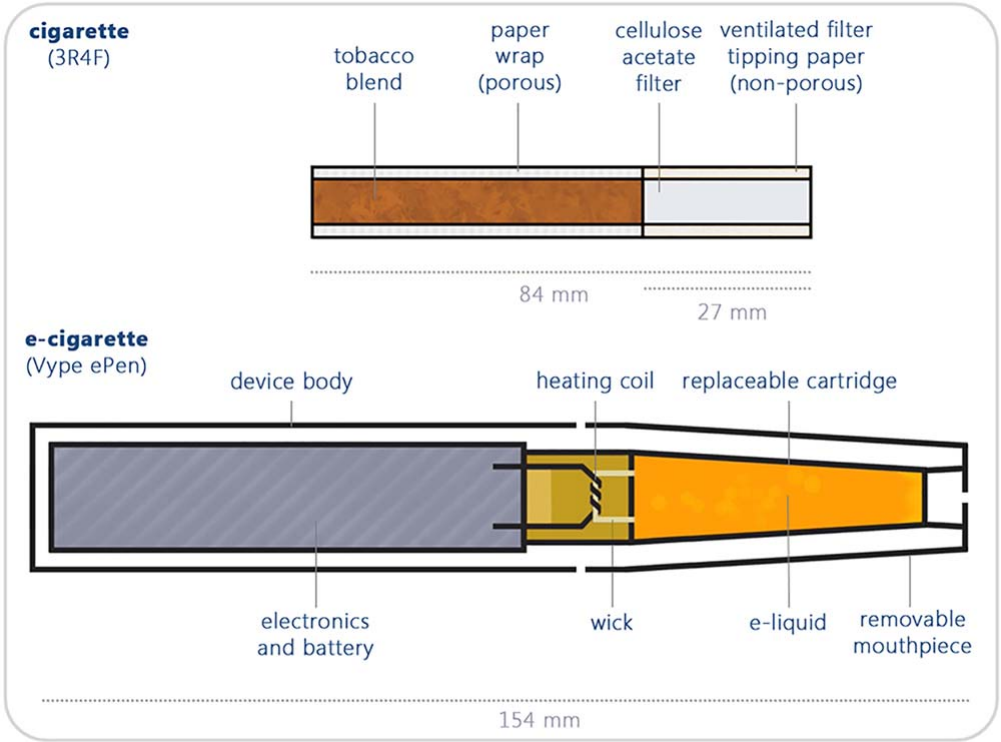
Schematic representation of Vype ePen (e‐cigarette) compared to the 3R4F reference cigarette [Adapted from Taylor et al. [2016]].

Vype ePen (Nicoventures, Blackburn, UK) e‐cigarettes were also used in this study. Vype ePen were obtained from Nicoventures Trading (www.govype.com). Vype ePen is a rechargeable, closed modular system, consisting of a rechargeable battery section and a replaceable cartridge (cartomizer) of nicotine‐containing liquid (e‐liquid; Fig. 1). It is operated by a button and has two voltage settings; 4 V and 3.6 V (4 V was used in the study). Vype ePen e‐liquid cartridges (Blended Tobacco Flavor) contained a reported 18 mg/mL nicotine and were stored at room temperature. Devices were used with fully charged batteries and loaded with a new cartomiser for each use.

### Generation of TPM and ACM

The production of 3R4F TPM was carried out on a Borgwaldt RM200A rotary smoking machine (Borgwaldt‐KC, Hamburg, Germany). Cigarettes were smoked under the HCI puffing regime (55 mL puff volume, of 2 sec duration, every 30 sec, 100% vent blocking) using a bell‐shaped puff profile, as per the Health Canada Official Method T‐115 [Health Canada, 1999 1999]. ACM from the e‐cigarette was produced using a Borgwaldt LM20X (Borgwaldt‐KC) linear machine with a modified version of the regime used for TPM production in which the puffing regime was 3 sec duration using a square‐wave puff profile (CORESTA, 2015; Table 1). The e‐cigarette was activated via the use of a puff‐synchronised robot, 1 sec prior to the puff commencing and was held in position for the duration of the puff.

**Table 1:** Aerosol generation regimens

Product	Puff regimen	Puff volume (mL)	Puff frequency (s)	Puff duration (s)	Puff profile	Vent blocking	Coil preactivation (s)
3R4F	HCI^a^	55	30	2	Bell	100%	N/A
ePen	CRM^b^	55	30	3	Square	N/A	0

a HCI T‐115 [Health Canada, 1999 1999].

b CRM No. 81 [CORESTA, 2015].

N/A, metric not applicable.

**Figure 2:**
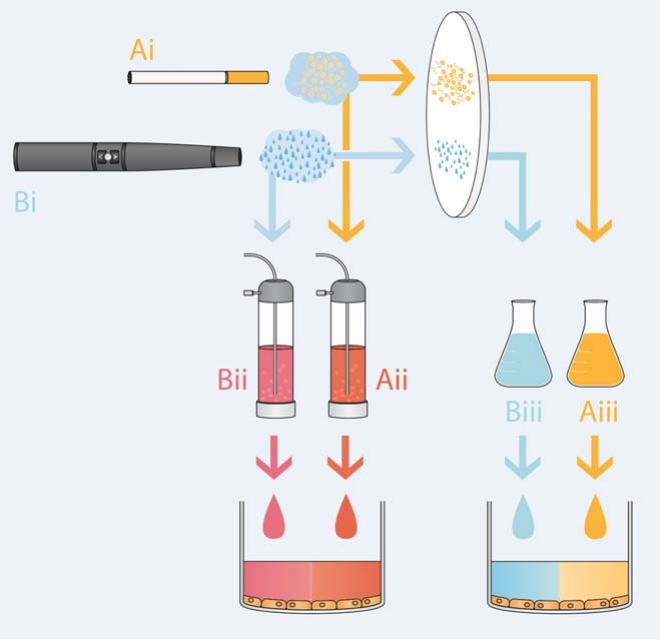
Schematic diagram of production of TPM/ACM and aerosol AqE from 3R4F (**Ai**) and ePen (**Bi**) aerosol emissions. AqE was prepared by capturing water‐soluble vapor and particulate aerosol constituents in cell culture medium using a glass impinger (**Aii** and **Bii**). TPM/ACM was generated by trapping aerosol matter on a Cambridge filter pad and then eluting this using DMSO (**Aiii** and **Biii**).

Approximately 150 mg of TPM or ACM were collected on 44 mm Cambridge filter pads (Whatman, Maidstone, UK). The same number of puffs was used to generate enough TPM/ACM from each product for each pad (4 sticks of 3R4F (10 puffs/stick) and 40 puffs from the e‐cigarette). Pads were weighed before and after TPM/ACM collection. Dimethylsulphoxide (DMSO) was used to elute the TPM or ACM from the pads to a stock concentration of 24 mg/mL (Fig. 2). Additional pads were prepared in parallel for determination of water and nicotine content in the TPM/ACM. A single batch each of 3R4F TPM and e‐cigarette ACM was created for all experiments, by pooling the extracts from a number of pads. The extracts were stored in single‐use volumes at −80°C. The samples were shipped on dry ice to the testing facility and stored at ≤–60°C until required.

### Generation of AqE

3R4F reference cigarettes and e‐cigarettes were smoked on a Cerulean SM‐450 linear smoking machine (Cerulean, VA) under the same regimes described above (Table 1).

AqE from both the 3R4F cigarette and e‐cigarette was produced by bubbling 10 puffs from each product through 20 mL of nonsupplemented Dulbecco's modified Eagle medium (DMEM)/F12 medium (Gibco, New York, NY) using a glass impinger (Fig. 2). To provide a sufficient volume of sample for cell transformation and parallel cell growth assays, multiple samples were combined and then aliquotted into the required volumes for each experiment. The samples were sealed in glass containers, frozen at −80°C and shipped overnight to the testing facilities. Samples were used within 48 hr of production. A separate batch of AqE was therefore prepared and used for each treatment timepoint in the preliminary cytotoxicity assay and promotion/parallel cytotoxicity experiments.

The following measurements were taken and used as a form of quality control for each 3R4F AqE sample produced: carbon monoxide produced during the AqE production, AqE nicotine concentration, and the optical density at 320 nm. These tests were performed as previously described [Taylor et al., 2016].

### Cell Culture

Bhas 42 mouse fibroblast cells were obtained from Hatano Research Institute, Food and Drug Safety Centre, Japan Cell Bank (Ochiai, Japan). Cells were cultured in MEM (Gibco) supplemented with 5% FBS and 50,000 U penicillin and 50 mg streptomycin. Three days prior to treatment with TPM, ACM, or AqE, Bhas 42 cells were maintained in DMEM/F12 (Gibco) supplemented with 5% FBS. This media was used to dilute the test solutions to concentrations to be used in each experiment.

### Cell Growth

The test samples were assessed in a cell growth assay to determine the concentrations for the promoter assay. Frozen stock cells were thawed and cultured in 20–50 mL of M10F in T‐75 flasks, at a volume of 10 mL per flask. 3 to 4 days later, the cells at approximately 70% confluence were trypsinized and resuspended in DF5F at 0.7–1 × 10^4^ cells/mL. The cell suspension was transferred at a volume of 10 mL to T‐75 flasks and incubated for 3 days. The cells at approximately 70% confluence were then trypsinized and suspended in DF5F at 7,000 cells/mL. The cell suspension was distributed into each well of six‐well plates at a volume of 2.0 mL (14,000 cells/well). After seeding, the six‐well plates were incubated at standard conditions. Wells for the vehicle and blank controls were also included.

Test article treatments were performed 4 days later. Treatment media pH was checked and adjusted if needed. Three wells per test article concentration, vehicle control, positive control, and blank control were treated for 3 days. The wells were fixed with methanol; the cells were stained with crystal violet solution for at least 15 min, rinsed with water, and air‐dried. The dye retained by the cells in each well was extracted with 2.0 mL of extraction solution (50% [v/v] ethanol with 0.02 mol/L HCl) by gentle shaking for at least 10 minutes, and the optical density of each extract was measured at a wavelength between 540 and 570 nm. Growth rates relative to the solvent/vehicle control culture were calculated. The doses for the promoter transformation (definitive) assay were selected with the highest dose having approximately 50% relative toxicity. In cases where the test article increased cell growth, the concentration with increased cell growth was used as the highest concentration for the definitive assay.

### Promoter Assessment

**Figure 3:**
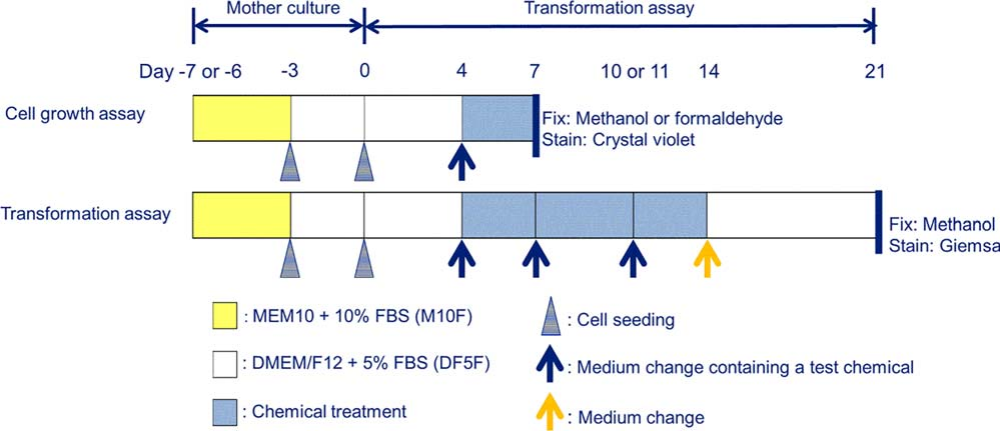
Schematic representation of cell growth assay and promoter transformation assay protocol used in this study [Adapted from OECD Guidance Document [Organisation for Economic and Cooperative Development, 2016]].

Prior to the initiation of promotion assay, a single cell growth assay, as illustrated in Figure 3, was carried out to identify concentrations with a relative toxicity of 50% and above. The promotion assay was carried out as shown in Figure 3 and described previously [Weisensee et al., 2013]. In brief, 14,000 cells/well were seeded and maintained in six‐well plates for 3 days (day 3 to day 0). A total of six wells per treatment were used. Cells were treated with test solutions for 10 days with a change to fresh treatment medium on days 7 and 10. On day 14, treatment medium was replaced with fresh DMEM/F12 supplemented with 10% FBS and left to incubate for a further 7 days, after which the cells were fixed with methanol. 12‐*O*‐Tetradecanoylphorbol‐13‐acetate (TPA; 50 ng/mL) served as a positive control. A test was considered valid if the positive control response was statistically significant compared to the vehicle control (t‐test, *P* ≤ 0.050. A cell growth assay was carried out in parallel to each promoter experiment as illustrated in Figure 3. A total of three wells per treatment group were used for this experiment.

### Data Analysis

A one‐sided ANOVA with Dunnett's test was used to assess the increase in the number of transformed foci per well. Significance level was set at 95% (*P* < 0.05). For the TPM and ACM experiments, the treatment groups were compared against the response of the DMSO vehicle control. AqE treatments were compared against cells treated with cell culture medium alone.

## RESULTS

### Nicotine Levels in Test Matrices

#### AqE

Mean nicotine levels of the three 3R4F AqE samples used in the promotion assay were approximately twice those of the e‐cigarette AqE (9.6 ± 0.5 and 4.7 ± 0.4 µg/mL, respectively; Supporting Information Table I). All QC measurements can be found in Supporting Information Table I.

#### TPM/ACM

Analytical chemistry data showed that 3R4F TPM contained 50 µg nicotine/mg, while e‐cigarette ACM contained 15.4 µg nicotine/mg (Supporting Information Table II).

### Promoter Activity of Cigarettes and e‐Cigarettes

#### AqE

A rangefinder experiment was used to ascertain the concentration of 3R4F AqE (0–50%) that could be used in the promoter and parallel cell growth assays (Supporting Information Table III). AqE concentrations of ≥15% resulted in a complete loss of cell viability. The following final concentrations were selected for use in the definitive promoter and parallel cell growth assays: 0.313, 0.625, 1.25, 2.5, 5, and 10% 3R4F AqE.

**Figure 4:**
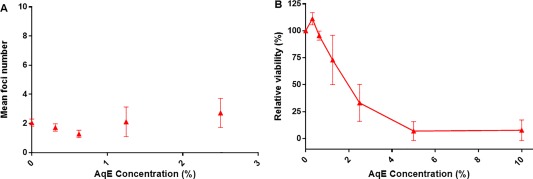
Results from the Bhas promoter cell transformation assay (**A**) and parallel cell growth assay (**B**) following 3R4F reference cigarette AqE treatment. Data are represented as the mean ± standard deviation of three independent experiments. Six wells were used for each treatment group in the promotion assay and three wells per treatment for the cell growth assay.

Concentrations of 2.5, 5, and 10% 3R4F AqE had high toxicity in the parallel cell growth assay, with a viability of less than 33% (Fig. 4B) and so were not scored for cell transformation. However, the transformation assay results from the lower noncytotoxic 3R4F AqE concentrations were negative, as they all failed to induce a significant increase in the number of transformed foci compared to the negative control across all three experiments (*P* ≥ 0.05, Figure 4A).

**Figure 5:**
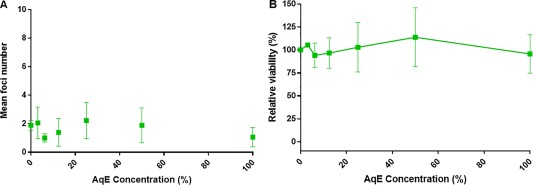
Results from the Bhas promoter cell transformation assay (**A**) and parallel cell growth assay (**B**) following ePen e‐cigarette AqE treatment. Data are represented as the mean ± standard deviation of three independent experiments. Six wells were used for each treatment group in the promotion assay and three wells per treatment for the cell growth assay.

AqE from ePen e‐cigarette was also assessed in a preliminary rangefinder (0–100% AqE) and found not to exhibit any toxicity at concentrations up to the maximum 100% AqE. It was determined to be negative for cell transformation in the definitive assay when tested up to this top concentration (Fig. 5). Positive control TPA treatment resulted in a significant increase in transformed foci in all experiments.

#### TPM/ACM

A preliminary concentration rangefinder was performed using concentrations of 12–120 µg/mL of TPM/ACM from each product. These experiments showed that the average relative viability in the 3R4F TPM treatment group did not fall below 50% even at the top concentration used (120 µg/mL, Supporting Information Table III). ACM from the e‐cigarette was less toxic than the TPM from the 3R4F cigarette, having low toxicity across all concentrations tested. The following final concentrations were selected for use in the definitive Bhas promoter and parallel growth assay: 3.0, 6.0, 12, 24, 48, 60, and 120 µg/mL TPM/ACM.

**Figure 6:**
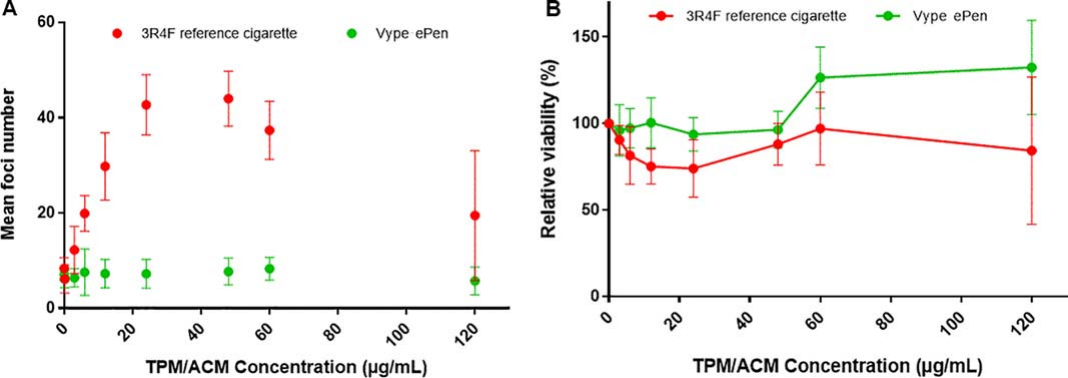
Bhas promoter cell transformation assay (**A**) and parallel cell growth assay (**B**) results from cells exposed to TPM from 3R4F cigarettes or ACM from ePen e‐cigarettes. Data are represented as the mean ± standard deviation of three experiments. Six wells were used for each treatment group in the promotion assay and three wells per treatment for the cell growth assay.

3R4F TPM was positive in all three experiments. Significant increases in the number of transformed colonies were consistently seen at concentrations of 6, 12, 24, 48, and 60 µg/mL across all three experiments (*P* > 0.05; Fig. 6A). ACM from the e‐cigarette was shown to be negative in all three promoter experiments. None of the tested concentrations caused a significant increase in the number of transformed colonies when compared to the vehicle control (*P* > 0.05; Fig. 6A).

ACM from the ePen e‐cigarette was shown to be less toxic than TPM from the 3R4F in the parallel cell growth assay (Fig. 6B). The average viability for the e‐cigarette ACM did not fall below 93.6 ± 9.8%, whereas the TPM from 3R4F did not fall below 73.9 ± 16.5%. Positive control TPA treatment resulted in a significant increase in transformed foci in all experiments.

## DISCUSSION

Cancer is a leading cause of death worldwide. In the UK, approximately 357,000 new cases were diagnosed in 2014 [Cancer Research UK, 2016a], while 163,444 people died from cancer in the same year [Cancer Research UK, 2016b]. Lung cancer accounts for the largest proportion (>1 in 5) of all cancer deaths in the UK, 2014 [Cancer Research UK, 2016b], and is also the most prevalent cancer type responsible for mortality globally, with 1.6 million deaths in 2012 [Ferlay et al., 2013]. A landmark study by Doll and Peto in 1981, recognized that most cancers in Western industrialised countries are attributable to lifestyle factors and habits such as cigarette smoking, diet, infections, and occupational chemical exposure, with diet (35%) and tobacco (35%) being the greatest single contributors [Doll and Peto, 1981]. It has been estimated that about 75% (smokers) or 50% (nonsmokers) of all cancer deaths in the United States could be avoided by eliminating these risk factors [Peto, 2001]. Reducing the exposure to the toxicants present in cigarette smoke is seen by many as a means of minimizing the burden of smoking related diseases such as cancer, both on society as a whole and on an individual level. The use of e‐cigarettes as a replacement for traditional cigarettes has been advocated by institutions such as the UK Royal College of Physicians, due to the reduced toxicant profile of the aerosols that these products generate [Royal College of Physicians, 2016].

The assessment of the carcinogenic potential of cigarette smoke has traditionally relied on in vivo inhalation and skin painting studies [Coggins, 2002; Walaszek et al., 2007]. These approaches are not ideal for many reasons. Lung tumors are notoriously difficult to induce in rodent models upon exposure to tobacco smoke due to physiological and behavioural interspecies differences when compared to humans [Hecht, 2005]. The skin painting model, where tobacco smoke tar is topically applied to the dorsal skin of a rodent, can also be questioned as to its relevance to human lung exposure. In addition, the time and cost implications make the use of animal models such as these unsuitable for screening products in large numbers, as well as being considerably unethical . This has led to the use of in vitro genotoxicity assays for tobacco product assessment and the increased development and application of in vitro assays for the testing of novel tobacco products and nicotine delivery devices. This drive to develop and adopt in vitro approaches for carcinogenic risk assessment has grown in parallel with a desire from the wider scientific community to replace the traditional rodent bioassay for both ethical and practical reasons.

In vitro CTAs have been used since the 1960s for the detection of carcinogens [Berwald and Sachs, 1963]. They are often used as a complement to a traditional genotoxicity test battery, due to their ability to detect both genotoxic and nongenotoxic carcinogens. In 2013, the EU Reference Laboratory for Alternatives to Animal Testing recommended that CTAs should be considered in combination with complementary information as part of a weight of evidence approach to identify substances with carcinogenic potential [EURL ECVAM, 2013 2016]. The Bhas 42 CTA has also been the subject of an OECD Guidance Document [Organisation for Economic and Cooperative Development, 2016], which helps to pave the way for scientific and regulatory community to apply the assay in this way. We have previously described the development and application of a two‐stage modification of the Syrian hamster embryo (SHE) CTA to assess initiation and promotion potential of cigarette smoke [Breheny et al., 2005]. While this assay was successful in identifying initiator and promoter activity of cigarette smoke TPM, the dose–response curve of the assay for positive controls and TPM was not sufficiently robust to facilitate product comparisons. This in in contrast to the Bhas 42 CTA, which gave a robust dose‐reponse to cigarette smoke TPM [Weisensee et al., 2013] and prompted our current investigation on its potential for product assessment and comparison purposes.

This study is the first to assess the cell transformation potential of an e‐cigarette aerosol and to compare it to that of a conventional cigarette. Both products were evaluated using two different test matrices, produced from aerosols which were generated under standardised conditions [Health Canada, 1999 1999; CORESTA, 2015]. We investigated the use of AqE as a test matrix in this assay as an approach to capture vapor phase components of the aerosols. In this approach, aerosols were bubbled through cell culture medium to capture the particulate and vapor‐phase water‐soluble components [Taylor et al., 2016]. AqE from both the reference cigarette and the commercial e‐cigarette were negative in the Bhas promoter assay. However, AqE from the 3R4F cigarette showed a high level of cytotoxicity, and there was a very narrow concentration range at which it could be assessed for cell transformation frequency. The maximum concentration at which it could be scored for foci formation was 1.25% 3R4F AqE, while e‐cigarette could be scored at concentrations up to the maximum 100% AqE tested. This indicates that the composition and relative levels of cigarette smoke aerosol constituents captured by the AqE are more likely to drive cytotoxicity than to induce the cellular and molecular events that lead to cell transformation. The toxicity profile of 3R4F AqE indicated that an extremely narrow concentration range could be used for assessment of cell transformation by this test article. Any potential promoting activity could be masked by the toxicity of 3R4F smoke components captured in the AqE. In a recent study we have shown that gaseous components of the cigarette aerosol, such as carbonyls, are captured quite effectively, while particulate constituents such as tobacco specific nitrosamines (TSNAs) are less efficiently retained in this aqueous matrix [Taylor et al., 2016]. In the same study, 3R4F AqE was demonstrated to induce significantly higher levels of oxidative stress in human lung cells than its e‐cigarette equivalent [Taylor et al., 2016]. Cytotoxicity and oxidative stress are likely to be driven at least to some extent by the carbonyls and other gaseous components of the AqE. Further refinement of the AqE dilutions used in the promotion assay may mitigate against the observed toxicity, but the authors were not convinced that a clear dose‐response with a dynamic range could be obtained using AqE. AqE was deemed not an ideal test matrix for assessing the in vitro promoting activity of aerosols from cigarettes, and therefore also not useful for product comparison purposes in this study.

As AqE was not found to be an effective matrix for the comparison of the transforming potential of cigarettes with e‐cigarettes, we built upon previous studies with cigarette smoke TPM [Weisensee et al., 2013], to investigate the use of this test article for product comparison purposes. Particulate based TPM trapping techniques were used, which have been extensively described for cigarette smoke assessment [DeMarini et al., 2008]. For e‐cigarette aerosol matter capture, a comparable technique to TPM was used, whereby an e‐cigarette aerosol was captured on a Cambridge filter pad and eluted with DMSO for testing (Thorne et al, 2016).

TPM from a 3R4F reference cigarette is a strong promoter in the assay and was positive at many concentrations in this range, as low as 6 µg/mL TPM (12.5 ng/mL nicotine). Conversely, we have shown that the particulate matter (ACM) from e‐cigarettes is negative in the Bhas 42 CTA when tested at concentrations up to 120 µg/mL (77 ng/mL nicotine). This study follows our own previous findings that the Bhas 42 CTA can distinguish between the potential promotion activity of a conventional cigarette and a tobacco heating product [Breheny et al., 2015], and supports the work of others who demonstrated the potential use of the assay in tobacco product assessment [Weisensee et al., 2013; Han et al., 2016].

It is long‐established that the particulate phase of cigarette smoke can drive tumor promotion in rodent skin painting studies [Roe et al., 1959]. It is thought that the skin tumor promoting effect of the smoke “tar” in this assay may be a result of specific expansion of initiated cells [Meckley et al., 2004]. A strong positive correlation between a compound's ability to induce sustained hyperplasia and its tumor‐promoting efficacy has been observed, although the role of the mutation(s) caused by the initiator is also considered to be very important [Smith et al., 2006]. Our findings in this study certainly point to the particulate phase of the cigarette smoke aerosol as the principal protagonist of the promotion stage in tumourigenesis, and support the preferred use of TPM/ACM in studies to evaluate and compare the promotion potential of tobacco and alternative nicotine delivery devices.

Results from this study suggest that e‐cigarettes may have reduced tumor promoter activity compared to conventional cigarettes and therefore may provide a safer alternative to cigarettes. Furthermore, the Bhas 42 assay can be used as a complementary test alongside the traditional genotoxicity assays applied in the carcinogenic assessment potential of tobacco and nicotine products. Use of CTA and other in vitro endpoints, along with clinical studies and consumer behavior data, can be included as part of a reduced risk product assessment framework such as that proposed by Lowe et al. [2015], to help assess the safety of current and novel tobacco and nicotine products.

## CONFLICT OF INTEREST

The authors are employees of British American Tobacco or BioReliance Corporation. BioReliance conducted all experimental work and were funded by British American Tobacco. Nicoventures, UK, is a wholly‐owned subsidiary of British American Tobacco.

## AUTHOR CONTRIBUTIONS

Oluwatobiloba Oke and Damien Breheny designed the study. Kamala Pant and other Bioreliance personnel carried out the experiments and analyzed the data. Damien Breheny, Oluwatobiloa Oke and Marianna Gaça prepared the manuscript draft and draft figures and tables. All authors approved the final manuscript.

## Supporting information

Supporting Information
