# Supplementary material for: Comparative tumor promotion assessment of e‐cigarette and cigarettes using the in vitro Bhas 42 cell transformation assay
Source: Environ Mol Mutagen. 2017 Apr 26;58(4):190–8. doi: 10.1002/em.22091 (PMC5435921; doi:10.1002/em.22091)
Supplement: Supplementary file 1 — Supporting Information [file EM-58-190-s001.docx]

**Supporting information**

**Supplementary Table I:** QC measurements from 3R4F and ePen AqE

|  | 3R4F | | | | ePen | | | |
| --- | --- | --- | --- | --- | --- | --- | --- | --- |
|  | Experiment | | | | | | | |
|  | Concentration rangefinder | Promotion /parallel cell growth assays | | | Concentration rangefinder | Promotion /parallel cell growth assays | | |
|  |  | 1^st^ Treatment | 2^nd^ Treatment | 3^rd^ Treatment |  | 1^st^ Treatment | 2^nd^ Treatment | 3^rd^ Treatment |
| Total volume (mL) | 40 | 45 | 45 | 45 | 40 | 45 | 45 | 45 |
| Puff Number  (puff/mL) | 0.525 | 0.509 | 0.519 | 0.501 | 0.5 | 0.5 | 0.5 | 0.5 |
| Carbon monoxide  [mg/cig] | 30.68 | 30.2 | 29.6 | 30.5 | N/A | N/A | N/A | N/A |
| UV-OD | 0.896 | 0.743 | 0.778 | 0.776 | N/A | N/A | N/A | N/A |
| Nicotine  [µg/mL] | 9.95 | 9.38 | 10.1 | 9.24 | 3.98 | 4.95 | 4.24 | 4.96 |

**Supplementary Table II:** QC measurements from 3R4F TPM and ePen ACM

| QC measurements per pad  (mean ± standard deviation) | Product | |
| --- | --- | --- |
|  | 3R4F (4 sticks) | ePen (40 puffs) |
| TPM/ACM (mg) | 145.0 ± 4.2 | 141.3 ± 5.9 |
| H_2_O (mg) | 43.3 ± 1.3 | 34.9 ± 3.0 |
| Nicotine (mg) | 7.3 ± 0.2 | 2.2 ± 0.0 |
| µg nicotine/mg TPM/ACM | 50.6 | 15.4 |

**Supplementary Table III**: Data from concentration rangefinder experiments used to identify concentrations to be used in the Bhas promoter and parallel cell growth assays

| **Study** | | | | | | | |
| --- | --- | --- | --- | --- | --- | --- | --- |
| **A: 3R4F vs ePen AqE** | | | | **B: 3R4F TPM vs ePen ACM** | | | |
| **3R4F AqE Concentration** | **Relative Viability compared to vehicle control** | **ePen AqE concentration** | **Relative Viability compared to vehicle control** | **3R4F TPM Concentration** | **Relative Viability compared to vehicle control** | **ePen ACM concentration** | **Relative Viability compared to vehicle control** |
| 0.5 % | 90.6 % | 1.0 % | 103.5 % | 12.0 µg/mL | 82.9 % | 12.0 µg/mL | 122.5 % |
| 1.5 % | 82.4 % | 3.0 % | 96.6 % | 24.0 µg/mL | 68.3 % | 24.0 µg/mL | 134.1 % |
| 5.0 % | 74.4 % | 10.0 % | 86.2 % | 48.0 µg/mL | 87.6 % | 48.0 µg/mL | 135.8 % |
| 15.0 % | -8.1 % | 30.0 % | 81.1 % | 60.0 µg/mL | 97.7 % | 60.0 µg/mL | 82.1 % |
| 50.0 % | -12.1 % | 100.0 % | 90.6 % | 120.0 µg/mL | 70.3 % | 120.0 µg/mL | 77.0 % |
